# Dextran-based Nanomicelle System with Directly Ester-bound Gadolinium Chelates as a Magnetic Resonance Imaging Contrast Agent for Tumor Detection

**Published:** 2020

**Authors:** Lirong Ma, Weiyu Yang, Dingmei Wu, Mengmeng Jiang, Xiuzhong Yao, Weiyu Weng

**Affiliations:** a *Engineering Research Center of Pharmaceutical Process Chemistry, Ministry of Education, School of Pharmacy, East China University of Science and Technology, Shanghai 200237, PR China. *; b *Department of Radiology, Zhongshan Hospital Affiliated to Fudan University, Shanghai 200032, PR China. *; c *Shanghai Key Laboratory of New Drug Design, East China University of Science and Technology, Shanghai 200237, PR China.*; 1 * M l. and Y w. contributed equally to this work.*

**Keywords:** Dextran, Nanomicelle system, Biocleavable gadolinium chelates, Tumor imaging, Elimination

## Abstract

There is a strong need to develop MRI contrast agents (CAs) with lower *in-vivo *retention, stronger signal enhancement, and more specific imaging. Here, we report a novel dextran (DEX)-based nanomicelle system as an MRI CA with superior tumor imaging and relatively short intravascular persistence. Gadolinium (Gd)-chelate (DTPA-Gd) was conjugated directly to DEX hydroxyl via a degradable ester bond. DEX-DTPA-Gd was then modified with dodecylsuccinic anhydride to obtain the amphiphilic derivative, 2-dodecylsuccinic acid (DSA)-grafted DEX-DTPA-Gd. Nanomicelles were prepared by dissolving DSA-DEX-DTPA-Gd in water using ultrasonication. The physicochemical properties, cytotoxicity, and MRI efficiency of the synthesized CA were evaluated. The synthesized DSA-DEX-DTPA-Gd self-assembled into nanomicelles with an average diameter of 67.80 ± 5.21 nm. Within the given Gd concentration range, DSA-DEX-DTPA-Gd and Magnevist® exhibited similar cytotoxicity. DEX-based CAs resulted in a greater contrast enhancement of T_1_-weighted signal intensity in the tumor region than Magnevist®, and the tumors were clearly defined for at least 3 h. Simultaneously, the ester bond in DSA-DEX-DTPA-Gd facilitated the elimination of Gd chelates, compared with the relatively more stable amide linker. The DEX-based nanomicelle system with directly ester-bound DTPA-Gd may serve as an MRI CA with superior tumor imaging and relatively rapid elimination.

## Introduction

Magnetic resonance imaging (MRI) is a powerful tool available for clinicians and scientists to acquire *in-vivo* images of anatomy and physiology. Images are produced based on the relaxation process of protons, the positively charged hydrogen atoms that are abundant in tissues containing water and fat (1). To improve diagnostic accuracy through enhanced image contrast, exogenous contrast agents (CAs) are often needed to alter the relaxation rate of the surrounding hydrogen atoms (2). Paramagnetic metal ions and superparamagnetic iron oxide have been employed as CAs, among which gadolinium (Gd)-based CAs are the most widely used (3). Currently, all Gd-based CAs approved by the FDA are low-molecular-weight Gd complexes (LMGCs) (4), but there are limitations related to rapid clearance and nonspecific distribution in the body (5).

To overcome the drawback of traditional LMGCs, numerous studies have focused on the development of macromolecular and nanoscale Gd complexes (MNGCs) over the past few years. MNGCs offer several advantages over LMGCs including prolonged circulation time, high relaxivities, and tumor targeting via enhanced permeability and retention effects (5, 6). However, the clinical applications of MNGCs has been hindered by biosafety concerns (7) because the prolonged circulation time increases the probability that highly toxic free Gd^3+^ ions will be released (8). Two strategies have been employed to decrease the potential toxicity of MNGCs: the design of novel ligands able to increase thermodynamic stability of Gd complexes in MNGCs (9) and the use of biodegradable MNGCs to facilitate the rapid clearance of Gd complexes (10). Compared with the design of novel ligands, the use of biodegradable MNGCs is a more practical approach as various degradable linkages such as ester, disulfide, and amide bonds, have been successfully used for the development of pro-drugs and biodegradable polymers (11).

Dextrans (DEXs) are polysaccharides that have been in routine clinical use for decades, and have recently been used for the delivery of drugs, proteins, and imaging agents (12). Various DEX-based Gd CAs have been described (13-21); however, almost all the studies focused on the development of DEX-based CAs with long intravascular retention by using relatively stable linkages, which may increase the risk of the release of free Gd^3+^ ions, as mentioned above.

Herein, we report a DEX-based nanomicelles system as an MRI CA with high relaxivities, superior tumoral signal enhancement, and relatively short intravascular persistence. Gd-chelates (DTPA-Gd) were conjugated directly to DEX hydroxyls via an easily degradable ester bond to facilitate Gd-chelate release and elimination (22). DEX-DTPA-Gd was then modified with dodecylsuccinic anhydride to obtain the amphiphilic derivative, 2-dodecylsuccinic acid (DSA)-grafted DEX-DTPA-Gd, self-assembled into nanomicelles in aqueous medium. The physicochemical properties, complex stability, and cytotoxicity of the novel CA were characterized. The MRI efficiency of the new CA was evaluated by using Magnevist® and DSA-DEX-*NH*-DTPA-Gd as references. DSA-DEX-*NH*-DTPA-Gd is a nanoscale CA, in which DTPA-Gd is linked to DEX hydroxyl via a relatively more stable amide linker.

## Experimental

*Materials*

DEX (average MW 20 kDa), diethylenetriaminepentaacetic acid dianhydride (DTPA-dianhydride), 4-dimethylaminopyridine (DMAP), anhydrous DMSO, and 3-(4,5-dimethylthiazol-2-yl)-2,5-diphenyltetrazolium bromide (MTT) were purchased from Shanghai Aladdin Bio-chem Technology Co., Ltd. (Shanghai, China). Dodecylsuccinic anhydride, gadolinium chloride hexahydrate (GdCl_3_·6H_2_O), fetal bovine serum (FBS), and Leibovitz’s L-15 medium were purchased from Shanghai Macklin Biochemical Co., Ltd. (Shanghai, China). Gadopentetate dimeglumine (Magnevist®) was obtained from Bayer Vital GmbH (Leverkusen, Germany). Human pancreatic cancer cell line SW 1990 was obtained from Cell Bank of the Chinese Academy of Sciences (Shanghai, China). All other reagents were of analytical grade.

Five-week-old female nude mice, with a body weight of 18 ± 3 g were purchased from Shanghai Slake Experimental Animal Co., Ltd., Shanghai, China. The animals were allowed free access to food and water throughout the study. Animal experimental protocols were approved by the Ethics Committee of Zhongshan Hospital affiliated to Fudan University. All animal studies were performed in accordance with the “National Institutes of Health Guide for the Care and Use of Laboratory Animals”.

*Synthesis of DEX-DTPA*

DEX-DTPA was synthesized following the procedure described in the literature (23), with some modifications. DTPA-dianhydride (3.31 g, 9.26 mmol) and DMAP (1.13 g, 9.26 mmol) were dissolved in 30 mL of dry DMSO, to which a solution of DEX (3.0 g, containing approximately 18.52 mmol glucose units) in DMSO (30 mL) was added. The mixture was stirred at 75 °C for 24 h, and dialyzed (MW cutoff of 3.5 kDa) in water for 5 days. The resulting solution was filtered and freeze-dried. The nitrogen content of the product was determined by elemental analysis, and then the substitution degree of DTPA in DEX-DTPA (DS_DTPA_) was calculated from the following equation.

$\frac{3\times {\mathrm{DS}}_{\mathrm{DTPA}}}{162+357\times {\mathrm{DS}}_{\mathrm{DTPA}}}=\frac{{\mathrm{Mol}}_{N}}{M_{\mathrm{sample}}}$

 Equ. 1

Where 162 is the molecular weight of dehydrated glucose, 357 is the molecular weight of DTPA-dianhydride, Mol_N_ is the measured molar number of nitrogen, and M_sample_ is the mass of the measured sample.

*Synthesis of DSA-DEX-DTPA*

DSA-DEX-DTPA was synthesized in accordance with the described procedure in the literature (24), but with some modifications. DEX-DTPA (1.60 g, containing approximately 6.37 mmol glucose units) was dissolved in 50 mL of dry DMSO, to which a solution of dodecylsuccinic anhydride (0.65 g, 0.24 mmol) in DMSO (10 mL) was added. The mixture was stirred at 75 °C for 24 h, and dialyzed (MW cutoff of 3.5 kDa) in water for 3 days. The resulting solution was filtered and freeze-dried. The DS of DSA in DSA-DEX-DTPA (DS_DSA_) was determined by using ^1^H NMR (D_2_O) analysis from the following equation (25).

${\mathrm{DS}}_{\mathrm{DSA}}=\frac{A_{{-\mathrm{CH}}_{3}}}{A_{-\mathrm{CHO\_}}\times 3}\times 100\%$

Equ. 2

Where A_-CH3_ is the intensity of the peak at δ 0.81 arising from the methyl group of DSA, and A_-CHO-_ is the intensity of the peak at δ 4.89 arising from the hydroxyl group of DEX.

*Synthesis of DSA-DEX-DTPA-Gd*

DSA-DEX-DTPA (0.90 g, containing approximately 0.85 mmol DTPA) was dissolved in 30 mL of water, and the pH value was adjusted to approximately 6.5 with NaOH (1 mol/L), followed by the dropwise addition of GdCl_3_·6H_2_O (356.1 mg, 0.96 mmol) in water (5 mL). The mixture was stirred at room temperature for 2 h, and dialyzed (MW cutoff of 3.5 kDa) in water for 3 days. The resulting solution was filtered and freeze-dried. The Gd^3+^ content of the product was measured by using inductively coupled plasma optical emission spectrometry.

*Synthesis of DSA-DEX-NH-DTPA-Gd*

The synthesis of DSA-DEX-*NH*-DTPA-Gd is presented in the supplementary materials.

*Preparation and characterization of self-assembled nanomicelles*

To prepare self-assembled nanomicelles, 100 mg of DSA-DEX-DTPA-Gd was dissolved in 10 mL of water, ultrasonicated for 15 min in an ice bath using a probe-type sonicator (JY92–2; Scientz, Ningbo, China), and filtered through a 0.22 μm microporous membrane. The critical micelle concentration (CMC) of the nanomicelles was determined by using pyrene as a fluorescent probe (26). The particle size and surface charge of the nanomicelles were determined by dynamic light scattering and electrophoretic mobility measurements using a Zetasizer Nano ZS90 (Malvern, Worcestershire, UK). Each measurement was performed in triplicate and presented as the mean ± standard deviation.

*Evaluation of complex stability of DEX-based CAs in serum*

The complex stability of the DEX-based CAs was described by the fractions of Gd released after incubation of the CAs in serum. The Gd dissociation was determined as previously described in details (27, 28) with some modifications. Briefly, DSA-DEX-DTPA-Gd and DSA-DEX-*NH*-DTPA-Gd were dissolved in FBS to obtain solutions with a Gd concentration of 1 mmol/L. To prevent microbial growth, gentamicin sulfate was added to the serum to obtain a concentration of 0.02%. Aliquots of 0.5 mL were withdrawn at the 15th day. The samples were transferred onto a metal chelate column (1-mL Chelating Sepharose column), which was then washed with 10 mL of Bis-Tris buffer (pH 6). Free Gd^3+^ ions were eluted with 20 ml of nitric acid (10 mmol/L) and subjected to ICP-OES analysis.

*Cytotoxicity assay*

The cytotoxicity of DSA-DEX-DTPA-Gd and Magnevist® was assessed by using the MTT assay. SW 1990 cells were seeded in 96-well plates with L-15 medium at a density of 1×10^4^ cells/well and allowed to adhere for 12 h prior to the assay. The cells were then treated with DSA-DEX-DTPA-Gd nanomicelles and Magnevist® to give final Gd^3+^ concentrations of 0.05-1.25 mmol/L in a total volume of 100 µL at 37 °C for 48 h. Untreated cells were used as controls. After incubation, the medium was discarded, the wells were thoroughly washed with cell culture medium, and 80 µL of fresh medium was added per well. Subsequently, 20 µL of MTT reagent (5 mg/mL) was added to each well. The plate was then incubated for another 4 h at 37 **ºC.** The medium was removed and 100 µL DMSO was added to each well to solubilize the formed formazan crystals. The optical density of the solution at a wavelength of 490 nm in each well was immediately determined. The percentage viability of the cells was calculated from the following equation.

$\mathrm{Cell visibility\%}=\frac{{\mathrm{OD}}_{\mathrm{sample}}-{\mathrm{OD}}_{0}}{{\mathrm{OD}}_{\mathrm{control}}-{\mathrm{OD}}_{0}}\times 100\%$

Equ. 3

Where OD_sample_ is the optical density of the sample wells, OD_control_ is the optical density of the control wells, and OD_0_ is the optical density of blank wells without cells.

*In-vitro relaxivity measurement*

*In-vitro* T_1_ relaxivities (r_1_) of Magnevist®, DSA-DEX-DTPA-Gd, and DSA-DEX-*NH*-DTPA-Gd were measured by using a Magnetom Verio 3.0 T MRI scanner equipped with a 12-channel receive-only head coil and Syngo MR B17 software (Siemens AG, Healthcare Sector, Erlangen, Germany). T_1_ was quantified using a 3D VIBE sequence with an echo time (TE) of 1.73 ms, repetition time (TR) of 400 ms, FOV of 300 mm, slice thickness of 1.5 mm, first flip angle of 2**°**, second flip angle of 14°, and voxel size of 0.5 × 0.4 × 1.5 mm^3^. The r_1_ value was calculated from the following equation (10).

$\frac{1}{{\left(T_{1}\right)}_{\mathrm{obsd}}}=\frac{1}{{\left(T_{1}\right)}_{w}}+r_{1}+[C]$

 Equ. 4

Where (T_1_)_obs_ is the observed longitudinal relaxation time of CA (ms), (T_1_)_w_ is the observed longitudinal relaxation time of water (ms), [C] is the concentration of Gd^3+^ (mmol/L), and r_1_ is the longitudinal relaxivity of CA (mM^−1^ s^−1^).

*In-vivo MR imaging*

Twelve nude mice were used for the *in-vivo* imaging study with three animals in each group. SW 1990 pancreatic cancer cells (1 × 10^6^) were injected into the pancreas of each mouse. MRI studies were initiated after tumor growth for 4 weeks. The diameter of the tumors is within the range of 5–12 mm. The mice were anesthetized with 1% nembutal sodium solution (50 mg/kg, i.p.). Magnevist®, DSA-DEX-DTPA-Gd, and DSA-DEX-*NH*-DTPA-Gd were dissolved in sterile saline to obtain solutions with a Gd concentration of 12.5 mmol/L. These solutions were administered to anesthetized mice via tail vein injection at a dose of 0.1 mmol/kg Gd^3+^. Mice were oriented in a supine position on the MRI system slider bed. T_1_-weighted MR images were acquired by using the Magnetom Verio 3.0 T MRI scanner equipped with a four-channel mouse coil, before injection and during the indicated time points. The following imaging parameters were used: TE of 12 ms, TR of 5.32 ms, FOV of 100 mm, slice thickness of 1.2 mm, and voxel size of 0.5 × 0.4 × 1.2 mm^3^. The MR images were analyzed by using Syngo MR B17. Regions of interest were manually set as the tumor, bladder, inferior vena cava, liver, heart, and kidney of each mouse. The data were presented as the change in R_1_, defined as 1/T_1_, from baseline (ΔR_1_).

## Results and Discussion

*Synthesis and characterization of DSA-DEX-DTPA-Gd nanomicelles*

**Figure 1:**
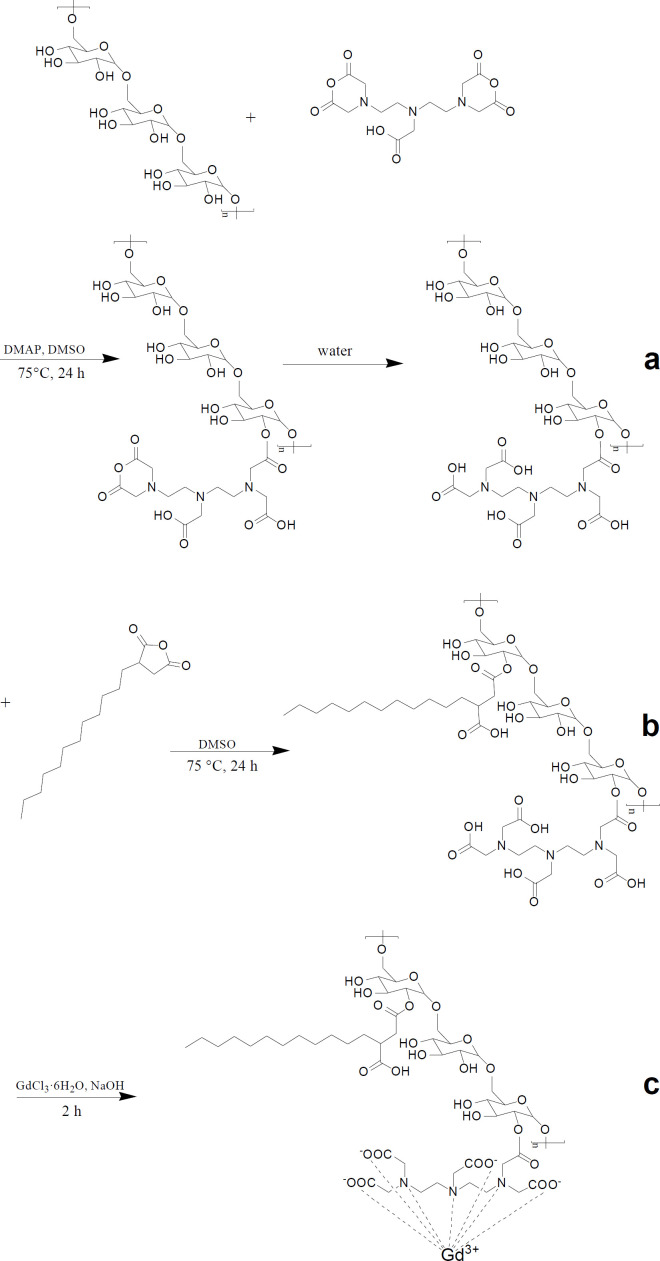
Schematic of MRI contrast agent synthesis. (a) Synthesis of DEX-DTPA. (b) Synthesis of DSA-DEX-DTPA. (c) Synthesis of DSA-DEX-DTPA-Gd

The synthesis of DSA-DEX-DTPA-Gd was performed in three steps (Figure 1). Firstly, DTPA was directly linked to DEX by an esterification reaction between the OH group of DEX and DTPA-dianhydride in anhydrous DMSO to form an ester bound, allowing the second unreacted anhydride ring to open through reaction with water (Figure 1a). Theoretically, the second anhydride ring can also react with another OH group of the DEX leading to the formation of a diester linkage (29). However, by conducting the esterification reaction in relatively dilute solution (23) and by decreasing the amount of DTPA-dianhydride added (29), the formation of the diester linkage will be most likely inhibited.

**Figure 2:**
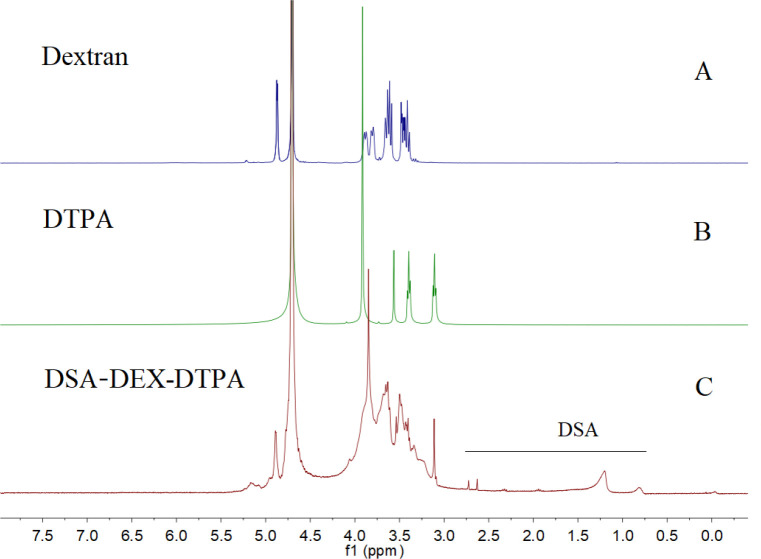
^1^H NMR spectra of A) DEX, B) DTPA, and C) DSA-DEX-DTPA in D_2_O

Subsequently, DSAs were introduced to the residual OH groups of DEX-DTPA by an esterification reaction using dodecylsuccinic anhydride as an esterifying agent (Figure 1b). The structure of dodecylsuccinic anhydride is similar to dodecenyl succinic anhydride, which has been frequently used in the modification of proteins and polysaccharides for versatile applications from pharmaceuticals to foods (30). The advantage of dodecylsuccinic anhydride over dodecenyl succinic anhydride is its chemical stability due to the saturated alkyl chain. Recently, dodecylsuccinic anhydride has been used in foodstuffs as an esterifying agent to modify starch (31). In the present study, dodecylsuccinic anhydride was used to synthesize amphiphilic DEX derivative for the first time. Because of the high reactivity of the succinic anhydride group, no catalyzer was needed for the esterification reaction. The ^1^H NMR (D_2_O) spectra of DEX, DTPA, and DSA-DEX-DTPA are shown in Figure 2.

Finally, Gd^3+^ was chelated to DTPA to obtain DSA-DEX-DTPA-Gd (Figure 1c). The content of Gd^3+^, and the DSs of DTPA and DSA of the product are shown in Table 1.

Nanomicelles were prepared by dispersing DSA-DEX-DTPA-Gd in distilled water using a probe-type ultrasonic treatment. The CMC value of DSA-DEX-DTPA-Gd was 27.4 μg/mL, implying that the synthesized DSA-DEX-DTPA-Gd can self-assemble into nanomicelles in aqueous solution. The Average hydrodynamic diameter, polydispersity index, and zeta potential of the nanomicelles are shown in Table 1. Generally, a PDI of ≤0.1 can be considered monodispersed, while systems with a PDI of 0.1–0.4 are considered moderately polydispersed (32). Therefore, both of the DEX-based CAs are moderately polydispersed nanomicelles.

*Complex stability of DEX-based CAs in serum*

Many attempts have been made to assess the stability of Gd-loaded CAs in biologic fluids, among which the assessment of Gd^3+^ dissociation in serum is one of the most accurate methods to reflect the *in-vivo* situation (27). After 15 days, almost identical amounts of the total Gd^3+^ (5.0% **±** 1.2% *vs* 4.5% ± 0.8%) had been released from DSA-DEX-DTPA-Gd and DSA-DEX-*NH*-DTPA-Gd, respectively. The result indicated that both DEX-based CAs were less stable than Magnevist (1.9% Gd^3+^ release after 15 days), but much more stable than those marketed nonionic linear Gd complexes (27).

*Cytotoxicity of DSA-DEX-DTPA-Gd*

**Figure 3:**
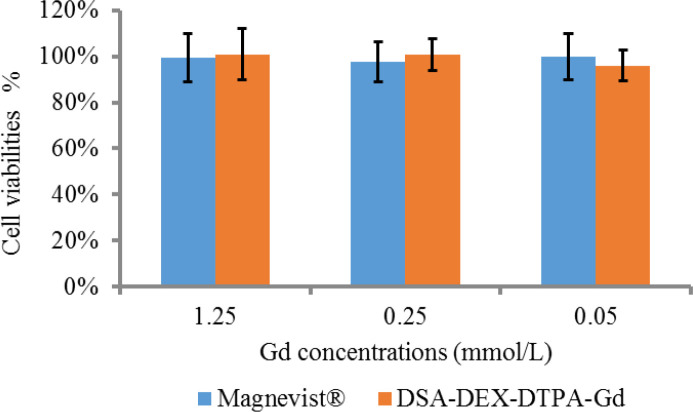
*In-vitro* cytotoxicity of DSA-DEX-DTPA-Gd and Magnevist® to SW 1990 cells in culture exposed for 48 h (n = 5).

The *in-vitro* cytotoxicity of DSA-DEX-DTPA-Gd was examined in SW 1990 cells by MTT assay compared with Magnevist®, the first approved intravenous CA for clinical imaging, as a reference. The recommended dose of Magnevist® for human is 0.1 mmol/kg and the human plasma volume is approximately 80 mL/kg, therefore, the maximum Gd concentration following intravenous injection is not more than 1.25 mmol/L. As illustrated in Figure 3, there was no statistically significant difference (*p *> 0.05) in the cytotoxic effects of DSA-DEX-DTPA-Gd and Magnevist® within the given Gd concentration range.

*In-vitro relaxivities*

The potential of DSA-DEX-DTPA-Gd as an MRI CA was evaluated by measuring its relaxivity (r_1_) using a 3 T MRI scanner. On a per Gd basis, DSA-DEX-DTPA-Gd has an r_1_ value of 9.42 mM^−1^s^−1^, which is superior to the r_1_ value of Magnevist® (5.25 mM^−1^s^−1^). The relaxivities of DSA-DEX-*NH*-DTPA-Gd was 11.20. The result confirmed that MNGCs present the advantage of high relaxivities over LMGCs. The enhanced r_1_ values for DEX-based CAs can be attributed to the slow global rotational motion of MNGCs that improves the relaxation effectiveness of each Gd^3+^ paramagnet (33).

*In-vivo tumor imaging*

Pancreatic cancer is the fourth most common cause of cancer death. Magnetic resonance imaging (MRI) is now a second-line modality for pancreatic cancer detection (34). CAs with stronger signal enhancement and more specific distribution can help improving the sensitivity of MRI for pancreatic cancer detection. The imaging efficiency of the DEX-based CAs was evaluated in SW 1990-bearing mice. T_1_-weighted images of the tumor region before and after injection of Magnevist®, DSA-DEX-DTPA-Gd, and DSA-DEX-*NH*-DTPA-Gd at a dose of 0.1 mmol/kg Gd^3+^ are shown in Figure 5. The temporal quantitative R_1_ changes relative to baseline (ΔR_1_) are shown in Figure 6. Before injection, the tumor tissue region showed low signal intensity on T1WI. After Magnevist® administration, a rapid and uneven enhancement was observed in the lesions. A significant contrast enhancement (ΔR_1_ ≈ 0.45 s^−1^) was observed in tumor periphery at 5 min, which then decreased rapidly. After the injection of DEX-based CAs, a significant and sustained ΔR_1_ elevation was observed at the tumor site. In the tumor boundaries, the increase in ΔR_1_ to a peak occurred within the time range of 30–60 min for DEX-based CAs, and the boundaries were clearly defined for at least 3 h (Figure 5). The prolonged enhancement duration will facilitate clinicians to obtain clear images and improve the diagnostic accuracy. These results demonstrate that fabricated DEX-based CAs are superior CAs for tumor MRI diagnostics.

DEX-based CAs also induced greater contrast enhancement in the tumor core than Magnevist® (Figure 6b); however, the tumor core displayed a smaller increase in R_1_ than the tumor periphery with all CAs. The preferential accumulation of CAs in the tumor periphery may be attributed to the elevated intra-tumoral interstitial fluid pressure that impedes the penetration of CAs into tumor tissues (35).

*In-vivo elimination of CAs*

The temporal quantitative ΔR_1_ profiles were used to reflect the *in-vivo* Gd^3+^ contents in the organs and blood, because R_1_ maps provide a good quantitative measure of the CA distribution in the whole body, and good agreement between the **Δ**R_1_ values and organ Gd^3+^ contents has been reported (23).

As DTPA-Gd is essentially eliminated via the renal excretion pathway (36), the MRI intensity accumulation in the bladder and the attenuation in the inferior vena cava post injection reflect Gd clearance from the blood. The temporal quantitative ΔR_1_ at bladder region after injection of Magnevist® and DEX-based CAs is shown in Figure 6c. Magnevist® is cleared rapidly, as evidenced by strong bladder signal enhancement with a ΔR_1_ of 123.78 s^−1^ at 30 min, then the intensity decreased gradually, which might be due to animal urination. After the injection of DSA-DEX-*NH*-DTPA-Gd, only a slight enhancement was observed in the bladder, with a sustained ΔR_1_ elevation of 4.45 s^−1^ at 3 h, which indicated slow clearance from the body. Compared with DSA-DEX-*NH*-DTPA-Gd, DSA-DEX-DTPA-Gd resulted in rapid and strong signal intensities in the bladder after injection with the measured peak ΔR_1_ of 20.59 s^−1^ at 2 h. This result provided supporting evidence that conjugating Gd-chelates directly to DEX hydroxyls with an easily degradable ester bond facilitated the renal excretion of Gd chelates.

The temporal quantitative ΔR_1_ in the inferior vena cava, which can reflect the profile of CAs in the blood following injection of Magnevist® and DEX-based CAs, is shown in Figure 6d. This result again confirms that the elimination rate of Magnevist® was much greater than that of DEX-based CAs, and that the ester bond between DTPA and DEX facilitated the elimination of Gd chelates.

*In-vivo organ imaging*

The ΔR_1_ profiles in the heart, liver, and kidney are presented in Figure 6e-g, respectively. Compared with Magnevist®, all DEX-based CAs produced superior and prolonged contrast enhancement in the aforementioned organs. These results demonstrated that fabricated DEX-based CAs were also superior for body imaging. Hals *et al*. (22) reported that the stability of DEX-based CAs with a direct ester-bound DTPA-Gd may be insufficient for use as macromolecular blood pool CAs. They suggested using amide bonds instead of the ester bond. However, the results of our research showed that DEX-based CA with directly ester-bound DTPA-Gd was a superior CA with good contrast enhancement and relative shortly intravascular persistence.

**Figure 4.**
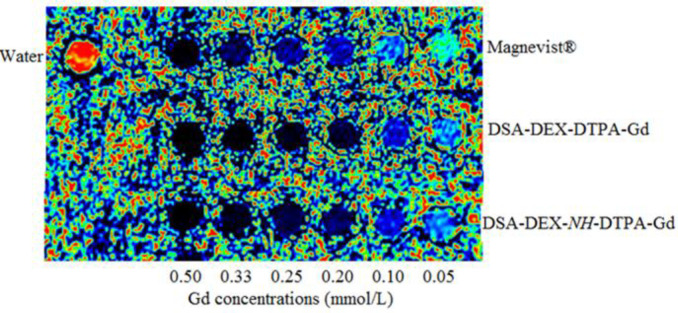
*In-vitro* T_1_-weighted MR pcolor (pseudo color) images of Magnevist®, DSA-DEX-DTPA-Gd, and DSA-DEX-*NH*-DTPA-Gd

**Figure 5:**
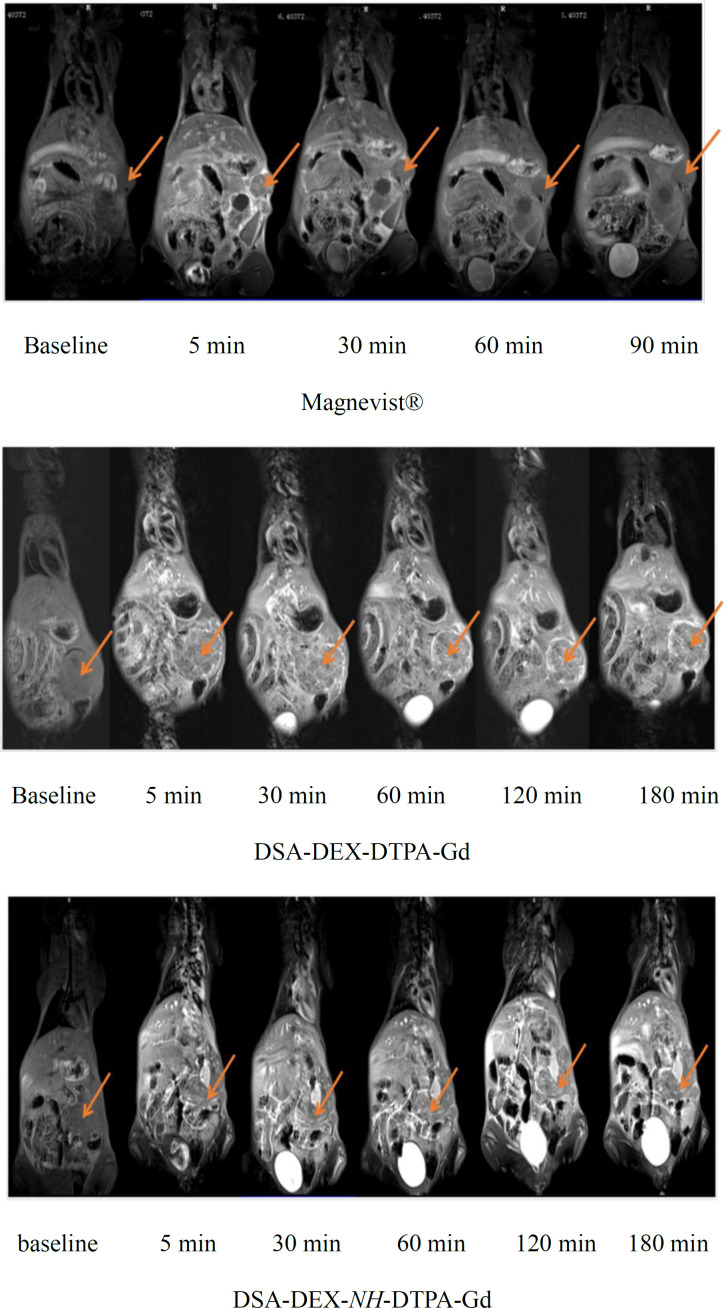
T_1_-weighted images of pancreatic tumors generated by using Magnevist®, DSA-DEX-DTPA-Gd, and DSA-DEX-*NH*-DTPA-Gd (arrows indicate *in-situ* pancreatic tumors).

**Figure 6:**
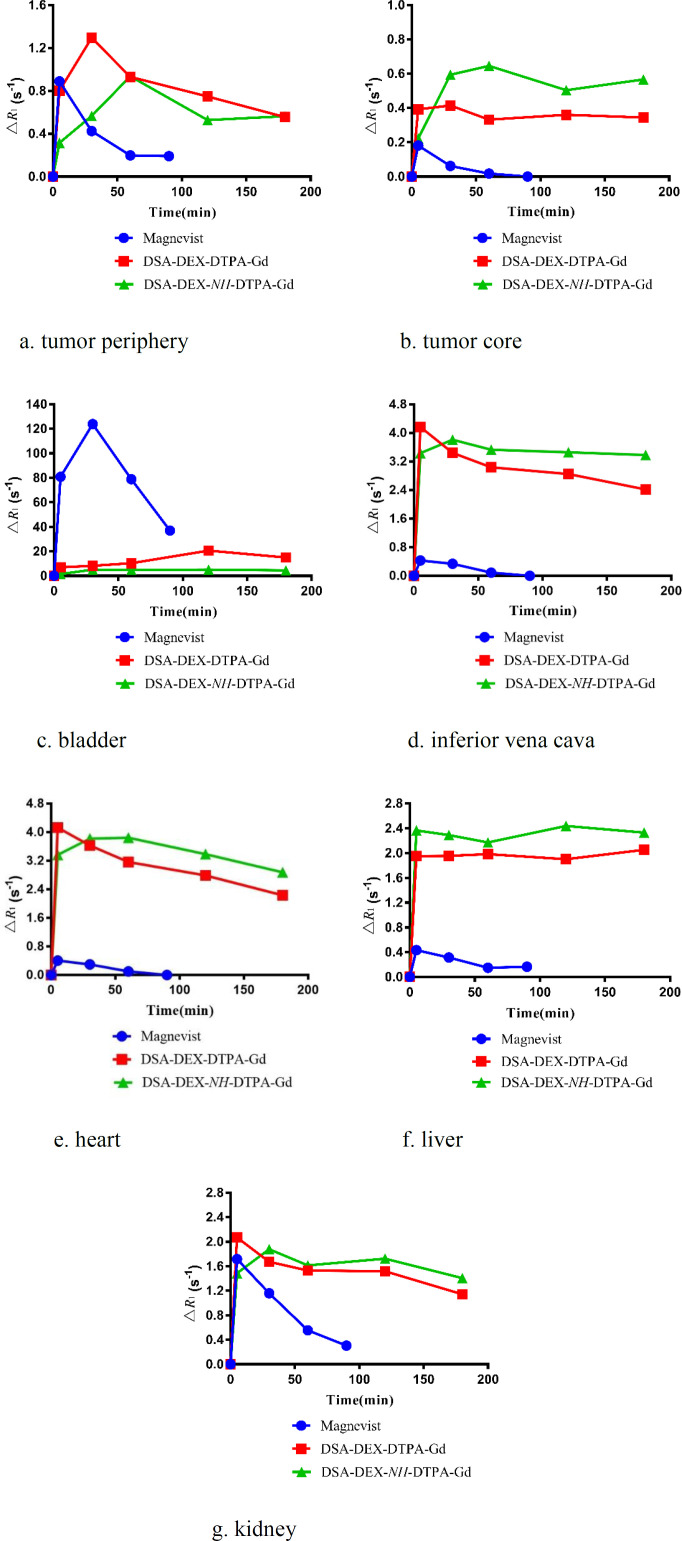
Change in relaxation rates, ΔR_1_ following intravenous injection of Magnevist®, DSA-DEX-DTPA-Gd, and DSA-DEX-*NH*-DTPA-Gd

**Table 1:** Physicochemical properties of DEX-based contrast agents

	**DSA-DEX-DTPA-Gd**	**DSA-DEX-** ***NH*** **-DTPA-Gd**
Gd content (%)	9.2	6.4
DSs of DTPA (%)	25.0	9.3
DS of DSA (%)	2.5	2.3
Hydrodynamic diameter (nm)	67.8 ± 5.2	96.2 ± 3.5
Polydispersity index (PDI)	0.35 ± 0.08	0.36 ± 0.11
Zeta potential (mV)	-6.8 ± 0.5	-2.9 ± 0.4

## Conclusion

In the present study, a novel DEX-based nanomicelle system was fabricated as an MRI CA with high relaxivities, superior tumoral signal enhancement, and relatively short intravascular persistence. Gd-chelates were conjugated to DEX hydroxyls with an easily degradable ester bond to facilitate Gd-chelate elimination, and hydrophobic DSAs were grafted to DEX to obtain the amphiphilic DEX derivative. The synthesized DSA-DEX-DTPA-Gd self-assembled into nanomicelles in aqueous medium. Compared with Magnevist®, DSA-DEX-DTPA-Gd exhibited superior T_1_ relaxivity and tumoral signal enhancement, reflecting the advantages of nanosized CAs. Meanwhile, compared with the relatively more stable amide linker, the ester bond in DSA-DEX-DTPA-Gd facilitated the renal excretion of Gd chelates, which could decrease the toxicity of Gd-based nanosized CAs. After comprehensive consideration of relaxivities, *in-vivo* persistence, and tumoral signal enhancement, we believe that DSA-DEX-DTPA-Gd is a potential CA for tumor MRI.
